# Identification of the PAK4 interactome reveals PAK4 phosphorylation of N-WASP and promotion of Arp2/3-dependent actin polymerization

**DOI:** 10.18632/oncotarget.20352

**Published:** 2017-08-18

**Authors:** Miao Zhao, Matthias Spiess, Henrik J. Johansson, Helene Olofsson, Jianjiang Hu, Janne Lehtiö, Staffan Strömblad

**Affiliations:** ^1^ Department of Biosciences and Nutrition, Karolinska Institutet, Stockholm, Sweden; ^2^ Cancer Proteomics Mass Spectrometry, Department of Oncology-Pathology, Science for Life Laboratory, Karolinska Institutet, Stockholm, Sweden

**Keywords:** p21-activated kinase 4, actin cytoskeleton, protein-protein interaction, mass spectrometry, VCA domain

## Abstract

p21-activated kinase 4 (PAK4) regulates cell proliferation, apoptosis, cell motility and F-actin remodeling, but the PAK4 interactome has not been systematically analyzed. Here, we comprehensively characterized the human PAK4 interactome by iTRAQ quantitative mass spectrometry of PAK4-immunoprecipitations. Consistent with its multiple reported functions, the PAK4 interactome was enriched in diverse protein networks, including the 14-3-3, proteasome, replication fork, CCT and Arp2/3 complexes. Because PAK4 co-immunoprecipitated most subunits of the Arp2/3 complex, we hypothesized that PAK4 may play a role in Arp2/3 dependent actin regulation. Indeed, we found that PAK4 interacts with and phosphorylates the nucleation promoting factor N-WASP at Ser484/Ser485 and promotes Arp2/3-dependent actin polymerization *in vitro.* Also, PAK4 ablation *in vivo* reduced N-WASP Ser484/Ser485 phosphorylation and altered the cellular balance between G- and F-actin as well as the actin organization. By presenting the PAK4 interactome, we here provide a powerful resource for further investigations and as proof of principle, we also indicate a novel mechanism by which PAK4 regulates actin cytoskeleton remodeling.

## INTRODUCTION

The six human p21-activated kinase (PAK) serine/threonine kinases are Rho family GTPase effectors that display a multitude of important functions in physiology and disease [1–5]. Among them, PAK4 is a Cdc42 effector that possesses critical functions in embryonic, neuronal and vascular development, immune defense and cancer [3, 4, 6–11]. PAK4 is overexpressed and genetically amplified in cancer cell lines as well as in cancer patients, including in lung, pancreas, ovary, prostate, breast and gastric cancer, leukemia, oral squamous-cell carcinoma and melanoma [3, 4, 12–20].

PAK4 regulates many cellular functions related to cancer progression, including cell proliferation and survival as well as cell morphology, adhesion and migration, which are dependent on the actin cytoskeleton [2, 11, 20–27]. The PAK4 regulation of cytoskeleton dynamics, cell adhesion and migration is in part mediated by interaction with and phosphorylation of PDZ RhoGEF [28, 29], GEF-H1 [30], paxillin [22], and integrin β5 [23, 24, 31]. PAK4 also interacts with and phosphorylates LIMK1 and the slingshot homologue SSH-1 [28–30], both part of a multi-protein complex with 14-3-3ζ, where PAK4 activates LIMK1, which in turn inactivates SSH1, leading to an increased ADF/cofilin activity that severs actin filaments and increases actin turnover [32]. However, actin turnover is tightly regulated by numerous additional actin binding proteins and complexes cooperating to ensure an optimal equilibrium between actin polymerization, depolymerization and monomer sequestration [33, 34]. Actin polymerization is initiated by actin nucleation mediated for example by formins for unbranched actin filaments and the actin-related protein 2/3 complex (Arp2/3 complex) for branched actin networks [35]. Nucleation promoting factors like the WASP/Scar family members regulate the Arp2/3 complex to control the protrusion of lamellipoda and filopodia [36, 37].

Most proteins execute their functions through their interaction with other proteins. Therefore, elucidating protein-protein interaction networks has become key to understand highly coordinated biological processes [38]. However, the so far reported PAK4 interactors might not be sufficient to explain the diverse cellular functions attributed to PAK4 and might not fully clarify the role of PAK4 in cell morphology, adhesion and migration. Further, no global characterization of the PAK4 interactome has yet been reported, we therefore employed isobaric tags for relative and absolute quantitation (iTRAQ) quantitative mass spectrometry (QMS) to identify and characterize the human PAK4 interactome.

The PAK4 interactome was enriched in known interactors, including the 14-3-3 family, but also revealed novel interactions such as most subunits of the chaperonin containing TCP-1 complex (CCT complex) and the Arp2/3 complex. Importantly, we found that PAK4 interacts with and phosphorylates N-WASP at Ser484/Ser485 and promotes Arp2/3-dependent actin polymerization. Consistent with this finding, PAK4 ablation impaired N-WASP Ser484/Ser485 phosphorylation, shifted the cellular equilibrium between globular actin (G-actin) and filamentous actin (F-actin) and altered the cellular F-actin organization. Taken together, we propose a novel mechanism by which PAK4 regulates the actin cytoskeleton and the PAK4 interactome provides a powerful resource for further investigations.

## RESULTS

### Identification of the PAK4 interactome by quantitative mass spectrometry

**Figure 1:**
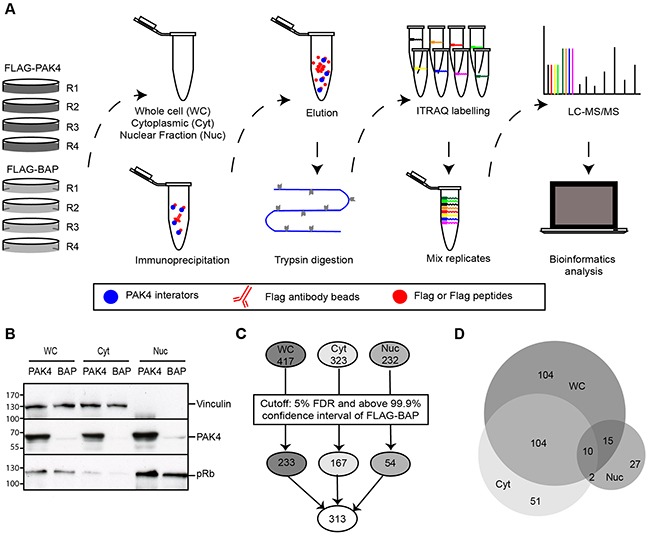
Identification of the PAK4 interactome by quantitative mass spectrometry **(A)** QMS workflow to identify the PAK4 interactome. MS analysis was performed using MCF7 cells stably expressing FLAG-PAK4 or FLAG-BAP (control). WC lysate and Cyt and Nuc subcellular fractions were analyzed in four independent biological replicates. After IP with anti-FLAG antibody and elution from FLAG beads with FLAG peptides, all samples were digested with trypsin and each labeled with a different iTRAQ 8-plex reagent. The eight iTRAQ labeled samples (four replicates of each FLAG-PAK4 and FLAG-BAP) were pooled and subjected to nano-LC-MS/MS analysis, followed by statistical and bioinformatic analysis. **(B)** Verification of subcellular fractionation. Lysates from WC, Cyt and Nuc fractions of FLAG-PAK4 and FLAG-BAP stably transfected MCF7 cells were analyzed by immunoblotting. Vinculin was used as a cytoplasmic marker; pRb as nuclear marker. **(C)** Schematic of the number of proteins identified by QMS in the different fractions before and after cut-off. Top: Total number of proteins recognized by QMS in each cellular fraction; Middle: Number of proteins in each fraction after cut-off; Bottom: Total number of unique proteins in all the fractions after cut-off. The cut-off criteria for specific FLAG-PAK4 associated hits was a combination of 5% FDR and above the 99.9% confidence interval of FLAG-BAP. **(D)** Venn diagram showing the number of specific PAK4 interacting proteins in WC and subcellular fractions.

To comprehensively characterize the PAK4 interactome, MCF7 cells stably expressing FLAG-PAK4 or FLAG-BAP (Bacterial Alkaline Phosphatase)(control) were subjected to fractionation and anti-FLAG immunoprecipitation (IP), followed by iTRAQ QMS, as illustrated in Figure 1A. From the two MCF7 cell lines, whole cell lysates (WC) were generated as well as two subcellular fractions: the cytoplasmic (Cyt) and nuclear (Nuc) fractions in order to enhance the number of identified proteins with subcellular resolution. The fractionation was verified by the enrichment of nuclear and cytoplasmic markers and PAK4 was present in substantial amounts in all the cellular fractions (Figure 1B). FLAG-PAK4 and FLAG-BAP (control) were immunoprecipitated in biological quadruplicates, trypsin digested and the samples were labelled with individual iTRAQ isobaric tags (8-plex iTRAQ) for quantification before analysis by nano-LC-MS/MS. Nano-LC-MS/MS identified in total 572 proteins; 417 in the whole cell lysate, 323 in the cytoplasmic fraction and 232 in the nuclear fraction at 1% false discovery rate (FDR) (Figure 1C). Among these proteins, 78% were identified with more than one peptide spectrum match (PSM) and 61 % of the proteins were identified by at least two unique peptides (Supplementary Figure 1A and 1B). We considered the PAK4 interactome as the proteins passing a combined cut-off of based on statistical testing (t-test with multiple hypothesis correction; p ≤ 0.05; 5% FDR); and FLAG-PAK4 signals above the 99.9 % confidence interval of the FLAG-BAP signal distribution. In total, 313 proteins passed our PAK4 interactome cut-off; 233 in the whole cell, 167 in the cytoplasmic fraction and 54 in the nuclear fraction (Figure 1C and Supplementary Table 1).

Among the 313 proteins, approximately two thirds of the cytoplasmic fraction and half of the nuclear fraction overlapped with the whole cell fraction, thereby each fraction also added additional, unique PAK4 interactors (Figure 1D). Previously known PAK4 interactors, including several 14-3-3 family proteins and a number of ribosomal proteins, were present in our dataset, validating our approach (Supplementary Table 1) [32, 39, 40]. Within a previously identified PAK1 interactome, we found 30 out of 71 proteins to overlap with the 313 PAK4 interactors found here [41] (Supplementary Table 1), suggesting that the PAK1 and PAK4 interactomes display a partial overlap.

### PAK4 interactome analysis reveals diverse cellular functions

**Figure 2:**
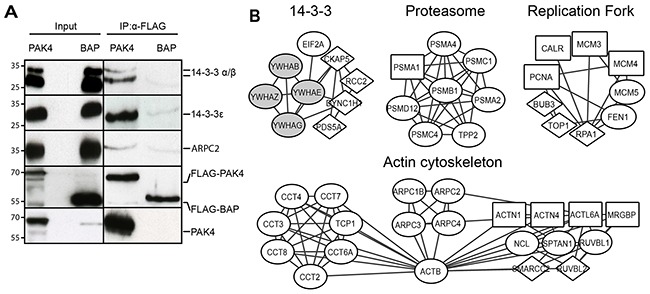
PAK4 interactome analysis reveals diverse cellular functions **(A)** Whole cell lysates derived from MCF7 cells stably expressing FLAG-PAK4 or FLAG-BAP were used for validation of QMS hits. After anti-FLAG IP and elution with FLAG peptides, samples were subjected to immunoblot analysis for the indicated proteins. Anti-FLAG (4^th^ row) and anti-PAK4 (5^th^ row) antibodies were used as controls. The left input panel shows immunoblotting of the two lysates. **(B)** PAK4 interactome networks obtained from the STRING database with the clusters identified by AutoAnnotate and visualized by Cytoscape. Diamond nodes: PAK4 interactors identified in the whole cell, or in both cytoplasmic and nuclear fractions or in all three fractions; Circle nodes: interactors identified in the cytoplasmic fraction or in both whole cell and cytoplasmic fraction; Squared nodes: interactors identified in the nuclear fraction or in both whole cell and nuclear fraction; Gray nodes: previously described PAK4 interactors.

We used immunoprecipitation from FLAG-PAK4 and FLAG-BAP whole cell lysates followed by immunoblotting to further validate our dataset. Firstly, we validated the previously known PAK4 interactors 14-3-3 α/β and 14-3-3 ε [26, 39]. We also validated the Arp2/3 complex subunit ARPC2, which is a novel PAK4 interactor (Figure 2A).

To analyze the protein-protein networks within the PAK4 interactome, we used the STRING database. We searched the PAK4 interaction network for highly connected regions including complexes and diverse cellular functions and named the identified clusters according to their functions (Figure 2B). These clusters included the actin cytoskeleton, 14-3-3, the replication fork and the proteasome. Ribosomal and ribonucleoproteins also formed clusters, but since these proteins have been described as common unspecific interactors [42], we did not follow up these further.

To strengthen the network analysis and assess other potential biological functions among the PAK4 interactors within our dataset, we performed a Gene Ontology (GO) analysis. First, we analyzed enrichment in cellular components and among others, we found the proteasome complex previously indicated to be associated with PAK4 [43], but also previously unknown PAK4 interacting components in form of the replication fork and the cytoskeletal regulatory Arp2/3 and CCT complexes (Supplementary Figure 2A and Supplementary Table 2). Pfam protein domain analysis displayed enrichment in the CCT chaperonin family, 14-3-3 as well as the proteasome and the minichromosome maintenance complex (MCM) (Supplementary Figure 2B and Supplementary Table 3). Together, the GO and Pfam terms overlapped well with the STRING network analysis showing that PAK4 may be involved in diverse cellular functions (Figure 2B, Supplementary Figure 2, Supplementary Table 2 and Supplementary Table 3).

### PAK4 associates with the CCT and Arp2/3 complexes

**Figure 3:**
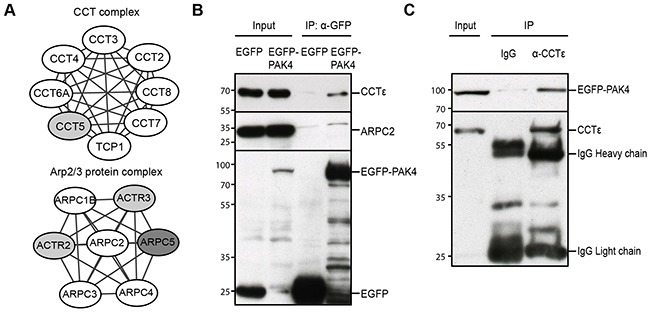
PAK4 associates with the CCT and Arp2/3 complexes **(A)** Several subunits of the Arp2/3 and CCT complexes were identified in the PAK4 interactome. White nodes: proteins passed QMS cut-off; Grey nodes: proteins appeared in MS but did not pass the QMS cut-off; Darker grey node: not in the MS list. **(B)** After GFP-Trap IP of H1299 cell lysates transiently expressing EGFP (control) or EGFP-PAK4, samples were subjected to immunoblot analysis for the indicated proteins. The upper panel is blotted with anti-CCTε, the middle panel with anti-ARPC2, while anti-GFP was used to control the IP efficiency in the lower panel. Input lanes are direct immunoblot of the used cell lysates. **(C)** After anti-CCTε IP of H1299 cell lysates transiently expressing EGFP-PAK4, blots were probed with an anti-GFP antibody in the upper panel. Anti-CCTε was used to control the IP efficiency in the lower panel. Input lane shows direct immunoblotting of the used lysate.

Within the actin cytoskeleton cluster (Figure 2B), we identified two complexes previously not recognized to be associated with PAK4: the CCT complex and the Arp2/3 complex. Importantly, seven of the eight subunits of the CCT complex and four of the seven subunits of the Arp2/3 complex were present in the PAK4 interactome (Figure 3A). To test if the PAK4 to Arp2/3 interaction was also detectable in a distinct cell line with a distinct tagging, we co-immunoprecipitated EGFP (control) and EGFP-PAK4 in transiently transfected H1299 cells and indeed EGFP-PAK4 co-immunoprecipitated ARPC2, whereas the EGFP control did not (Figure 3B). In addition, EGFP-PAK4 co-immunoprecipitated CCTε (Figure 3B). Also, an anti-CCTε monoclonal antibody (mab), but not IgG control, co-immunoprecipitated EGFP-PAK4 (Figure 3C). The associations between these subunits and PAK4 in combination with the presence of multiple subunits in the interactome indicate that PAK4 associates with the CCT and Arp2/3 complexes.

### PAK4 interacts with and phosphorylates N-WASP

**Figure 4:**
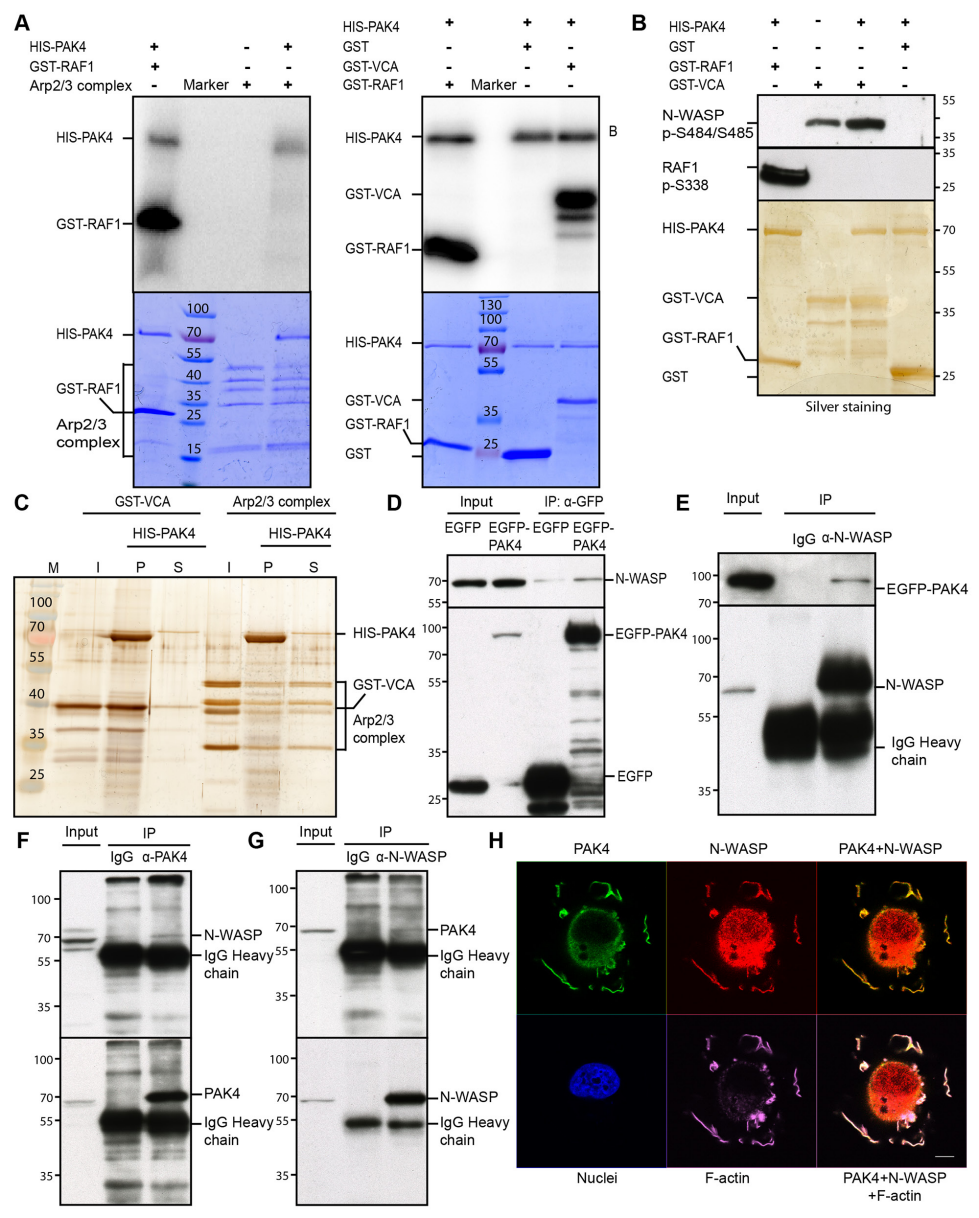
PAK4 interacts with and phosphorylates N-WASP **(A)** PAK4 mediated phosphorylation was analyzed by an *in vitro* kinase assay using recombinant HIS-PAK4 together with the Arp2/3 complex (left panel) or GST-VCA (right panel) as substrates, with GST as a negative control and GST-RAF1 (332–344) as a positive control (upper panels). The lower panels display the protein loading in the assays by Coomassie Brilliant Blue staining. **(B)** HIS-PAK4 phosphorylation of the WASP VCA domain was analyzed using an anti-N-WASP pSer484/Ser485 antibody after a kinase assay using recombinant HIS-PAK4 with GST-VCA as a substrate. GST serves as a negative control, while the anti-RAF1 pSer338 antibody was used as a positive control to detect GST-RAF1 phosphorylated by PAK4 (upper panel). The lower panel shows the loading of HIS-PAK4 protein and GST-fusion proteins used in the assay by silver staining. **(C)** HIS-PAK4 was pulled-down in the presence of GST-VCA or the Arp2/3 complex with Ni-NTA agarose and input (I), supernatant (S) and pellet (P) analyzed by silver staining. **(D)** IP of EGFP control or EGFP-PAK4 transiently expressed in H1299 cells analyzed by immunoblotting using an anti-N-WASP antibody (upper panel right two lanes). The left two lanes show immunoblotting of the input lysates. Anti-GFP was used to control the expression and IP efficiency in the lower panel. **(E)** N-WASP was immunoprecipitated with an anti-N-WASP antibody from lysates of H1299 cells transiently expressing EGFP-PAK4 and samples were analyzed by immunoblot using an anti-GFP antibody with the lysate input to the left (upper panel). Anti-N-WASP was used to control the expression and IP efficiency in the lower panel. **(F)** PAK4 was immunoprecipitated with an anti-PAK4 antibody from lysates of MCF7 cells, with rabbit IgG as a control, samples were analyzed by immunoblot using an anti-N-WASP antibody with the lysate input to the left (upper panel). Anti-PAK4 blotting was used to control IP efficiency in the lower panel. **(G)** N-WASP was immunoprecipitated with an anti-N-WASP antibody from lysates of H1299 cells, with rabbit IgG as a control, samples were analyzed by immunoblot using an anti-PAK4 antibody with the lysate input to the left (upper panel). Anti-N-WASP blotting was used to control IP efficiency in the lower panel. **(H)** PAK4, N-WASP and F-actin co-localized in the cell periphery after re-plating. FLAG-PAK4 was labeled with an anti-FLAG mab (Green), N-WASP with an anti-N-WASP antibody (Red), F-actin with SiR-actin (Purple) and Nuclei with Hoechst (Blue), Scale bar: 10 μm.

Given that the PAK family member PAK1 was reported to phosphorylate ARPC1B [44], we examined if PAK4 may phosphorylate any subunit of the Arp2/3 protein complex. To this end, we performed an *in vitro* kinase assay using purified, recombinant PAK4 and Arp2/3 protein complex together with [γ-32P] ATP. However, we did not detect any phosphorylation of the Arp2/3 complex subunits (Figure 4A, left). In addition to the Arp2/3 complex, we also tested the VCA domain from WASP, a VCA domain that is highly conserved among the WASP family proteins that interacts with and activates the Arp2/3 complex [45]. Interestingly, we found PAK4 phosphorylation of the VCA domain (Figure 4A, right). In order to identify possible phosphorylation site(s) in the VCA domain in N-WASP, which is the Arp 2/3-interacting VCA domain protein expressed in the cancer cells here used, we used the PhosphoSitePlus online tool [46], revealing Serines 484 and 485 as the most frequent sites in the N-WASP VCA domain. To test if PAK4 may phosphorylate these serines, we used a phospho-specific Ser484/Ser485 N-WASP antibody, revealing that PAK4 phosphorylated the VCA domain at the corresponding sites (Figure 4B).

Further, we tested if the association between PAK4 and the VCA domain and/or the Arp2/3 complex were direct interactions by performing *in vitro* pull-down assays using purified proteins (Figure 4C). Indeed, we found a strong enrichment of GST-VCA in the PAK4 pellet whereas the Arp2/3 complex remained mostly in the supernatant. These data suggest that PAK4 directly interacts with the VCA domain *in vitro* and therefore that the association by co-IP between PAK4 and the Arp2/3 complex might be indirect.

To assess the interaction between PAK4 and N-WASP *in vivo*, we used H1299 cells co-transfected with EGFP control or EGFP-PAK4 and found that while EGFP-PAK4 co-immunoprecipitated N-WASP, EGFP did not (Figure 4D). Also, the reverse co-IP using an anti-N-WASP antibody, but not the IgG control, co-immunoprecipitated EGFP-PAK4 (Figure 4E). Importantly, endogenous PAK4 co-immunoprecipitated endogenous N-WASP and endogenous N-WASP co-immunoprecipitated endogenous PAK4 (Figure 4F-4G). PAK4 and N-WASP also co-localized at F-actin bundles and leading edges at the cell periphery of MCF7 cells re-plated onto collagen type I (Figure 4H).

### PAK4 promotes actin polymerization and alters cellular actin organization

**Figure 5:**
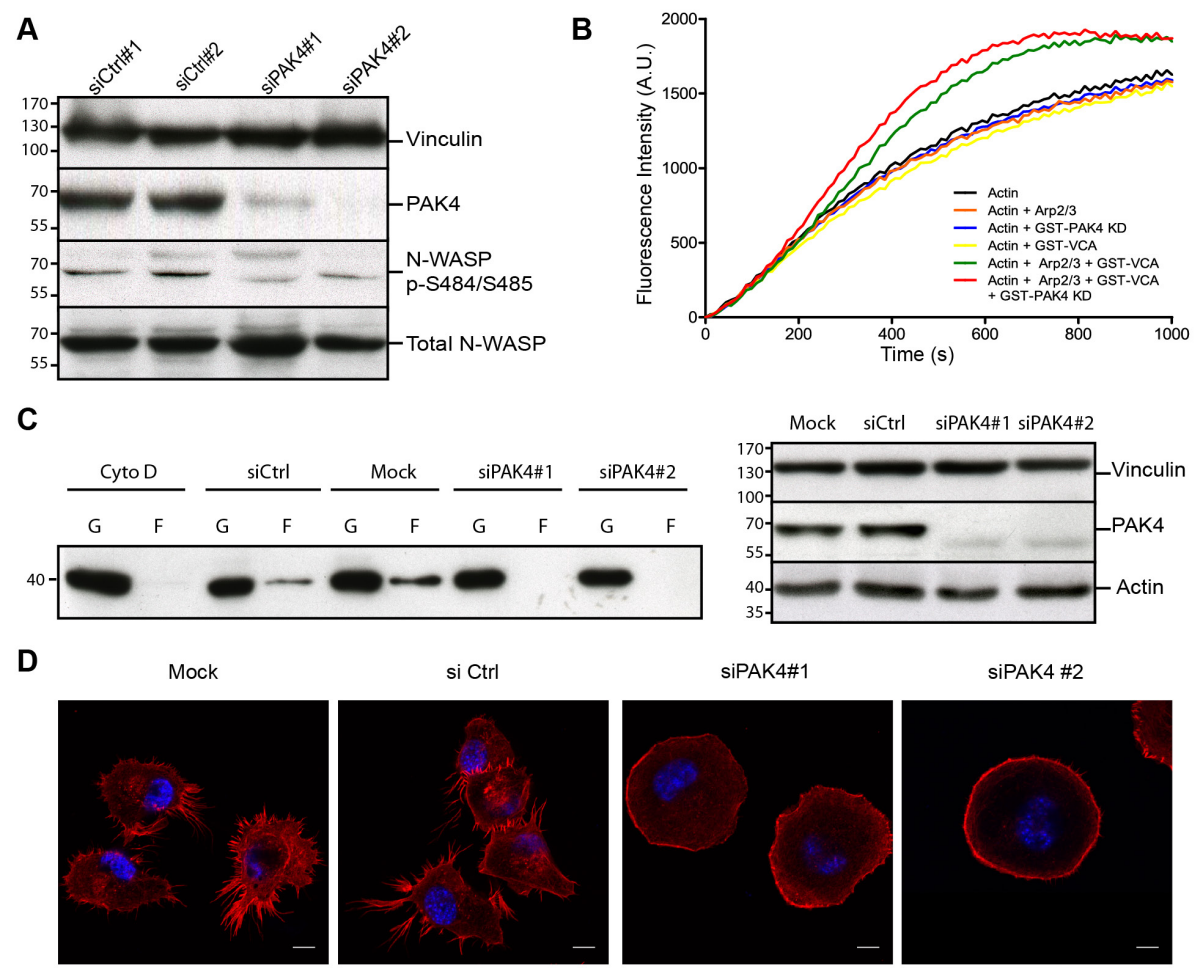
PAK4 promotes actin polymerization and alters cellular actin organization **(A)** Immunoblotting of lysates from control siRNA transfected cells and PAK4 knockdown (siPAK4) cells with anti-N-WASP and anti-N-WASP pSer484/Ser485 antibodies. PAK4 knockdown efficiency was detected with an anti-PAK4 antibody and vinculin was used as a loading control. **(B)** Actin polymerization reactions were performed with actin, Arp2/3 complex, GST–VCA and GST-PAK4 KD (kinase domain) in different combinations as indicated. The amount of polymerized actin over time is indicated by the increase in fluorescence intensity. **(C)** G-actin and F-actin were separated by centrifugation in H1299 cell lysates with or without PAK4 siRNA-mediated knockdown. Left panel: Cytochalasin D (Cyto D) treatment was used as a control to block actin polymerization. Immunoblot analysis with an anti-actin antibody shows the amount of G-actin (G) and F-actin (F) for each condition. Right panel: PAK4 knockdown efficiency was assessed by immunoblotting using vinculin as a loading control (top). In addition, the total amounts of actin were analyzed (bottom). **(D)** siRNA knockdown of PAK4 alters the cellular morphology and F-actin distribution in H1299 cells. Nuclei were stained with Hoechst (Blue) and F-actin with Phalloidin (Red). Scale bar: 10 μm.

To test if PAK4 affects phosphorylation of N-WASP *in vivo*, we analyzed whole cell extracts in the presence or absence of PAK4 with the phospho-specific Ser484/Ser485 N-WASP antibody. The N-WASP Ser484/Ser485 phosphorylation decreased upon PAK4 depletion by siRNA (Figure 5A). Phosphorylation of the VCA-domain has been reported to increase Arp2/3 dependent actin polymerization and we therefore hypothesized that PAK4-mediated phosphorylation might have a similar effect [47]. We used an *in vitro* actin polymerization assay and as reported, the Arp2/3 complex or VCA domain alone had no effect, but when combined they increased actin polymerization [48]. Importantly, pre-incubation of the VCA domain with the PAK4 kinase domain further increased actin polymerization while the PAK4 kinase domain alone had no effect on actin polymerization (Figure 5B). We here used recombinant PAK4 kinase domain because it displayed a more efficient phosphorylation of the VCA domain than full-length PAK4 (Supplementary Figure 3A).

Given the PAK4-mediated VCA-domain phosphory-lation and the increased VCA-Arp2/3 dependent actin polymerization, we tested if PAK4 may influence the equilibrium between F- and G-actin in H1299 cells. By ultra-centrifugation, we fractionated G-actin to the supernatant and F-actin to the pellet in whole cell extracts and immunoblotted against actin. Interestingly, while knockdown of PAK4 did not change the total amount of actin, the balance between G- and F-actin was shifted towards G-actin with markedly less F-actin detected in the pellets (Figure 5C). Having identified a shift in the G- to F-actin distribution, we labeled F-actin with phalloidin in H1299 cells in the presence or absence of PAK4 siRNA. While the control cells predominantly displayed an irregular shape and formed filopodia, the PAK4 knock-down cells appeared more round with the phalloidin labeling enriched at the cell periphery, where the F-actin formed a cortical actin ring (Figure 5D). Together, our results suggest that PAK4 dependent phosphorylation of N-WASP might promote actin polymerization, which is crucial for the cellular actin organization.

## DISCUSSION

We here report for the first time a comprehensive PAK4 interactome revealing a novel interaction with the Arp2/3 complex, a well characterized actin nucleator. While PAK4 is known to play a role in the regulation of the actin cytoskeleton, affecting cell morphology, adhesion and directed cell migration [2, 3], we propose a novel mechanism by which PAK4 fine-tunes actin polymerization. Our results demonstrate that PAK4 directly interacts with and phosphorylates the N-WASP VCA-domain. *In vitro,* we identified the phospho-site Ser484/Ser485 of the VCA domain to be phosphorylated by PAK4 accompanied by increased *in vitro* actin polymerization, while down-regulation of PAK4 *in vivo* decreased N-WASP Ser484/Ser485 phosphorylation and altered the F-actin pattern and the cell shape. Supported by these findings, we hypothesize that PAK4 phosphorylation of N-WASP contributes to Arp2/3-dependent actin polymerization and that lack of this phosphorylation contributes to change the cells from actin polymerization dependent cell surface protrusions, such as filopodia, to a more rounded shape with a cortical actin ring. Consistent with our hypothesis, casein kinase II was shown to phosphorylate WASP Ser483/Ser484 and thereby increase actin polymerization *in vitro* [47]. Also, filopodia formation was reported to be PAK4 dependent [11, 30] and we and others have previously shown that PAK4 promotes cancer cell migration [22–24, 26, 27, 30, 31, 49–51]. Given our new data, the role of PAK4 in cell migration and cytoskeleton regulation may in part be brought about by the PAK4-mediated N-WASP phosphorylation that could increase actin polymerization rates at the leading edge, finally resulting in increased cell migration. However, cell migration is a highly regulated process affected by a multitude of different factors [52]. For example, PAK1 can phosphorylate the Arp2/3 complex subunit ARPC1B, which is required for cell motility [44]. Also PAK4 regulates actin dynamics and cell motility by several pathways, including through interaction with and phosphorylation of GEF-H1, paxillin, integrin β5, LIMK1 and SSH-1 [21–23, 26, 27, 30, 50]. However, at this stage, it remains elusive how PAK4 may help balance the interplay between actin polymerization and depolymerization at the cell leading edge to achieve rapid cell migration, which will require further investigations. In addition, we report a novel interaction between PAK4 and the CCT complex, a chaperonin that is essential for folding of the cytoskeletal proteins actin and tubulin and that is overexpressed in cancer (just like PAK4) [53, 54]. Several CCT subunits have also been shown to regulate the actin cytoskeleton and the formation of cell surface protrusions [55, 56]. However, whether the CCT complex is a PAK4 substrate and if their association may be involved in regulation of the actin cytoskeleton remains to be examined.

Additionally, by the comprehensive characterization of the PAK4 interactome, we here provide a substantial number of new leads for future studies of the roles and molecular functions of PAK4. Firstly, our data suggest that PAK4 is involved in more cellular processes and protein complexes than previously appreciated. Secondly, we have identified a large number of so far unrecognized PAK4 interactors. Our approach involved a combined analysis of whole cell extracts and two subcellular fractions (cytoplasm and nucleus), and the inclusion of the subcellular fractions enhanced the number of identified PAK4-associated proteins by an additional 80 hits. Importantly, we were able to identify a substantial number of previously known PAK4-associated proteins, including the 14-3-3 protein family, giving confidence in our dataset.

The highly conserved 14-3-3 proteins are involved in regulating cell proliferation, differentiation, signal transduction, adhesion, survival and apoptosis [57]. Because the 14-3-3 proteins are multi-functional scaffold proteins, they may link PAK4 to various cellular functions.

Previous studies suggest that PAK4 is involved in controlling cell proliferation [5], possibly by promoting the G1-phase through decreased p21^Cip1^ protein levels [58] and/or the G2/M phase through phosphorylation of Ran-Ser135 [59]. Moreover, PAK4 was reported to be required for spindle positioning during mitosis [60] and to control proliferation through phosphorylation of ER-alpha [61]. Together, this indicates that PAK4 contributes to increased proliferation by promoting cell cycle progression. Here, we found several key proteins of the DNA replication fork in the PAK4 interactome, suggesting that PAK4 may regulate the DNA replication fork, adding complexity to the role of PAK4 in cell proliferation.

Another potential function of PAK4 interacting proteins is to control PAK4 activity and stability. The ubiquitin-proteasome system and the lysosomes are common ways to degrade proteins [62]. We found a large number of proteasome subunits in the PAK4 interactome, which is consistent with the recent report that PAK4 can be ubiquitinated and degraded by the proteasome [43].

Combining IP and MS is a widely used technique to study protein-protein interactions [38]. It is critical here to use proper controls and cut-off settings to, as good as possible, avoid false positive and false negative hits, although this remains a challenge. Notably, proteins of the ribosome, spliceosome and ribonucleosome were enriched in the PAK4 interactome. These are potential candidates for false positive hits [38], yet PAK4 was also shown to play a role in translation [40, 63–65]. We therefore have chosen to report these as well as all other hits passing our set cutoff. On the other hand, even by the use of fractionation, we still cannot expect to detect all PAK4 interactors. For example, we did not detect the known PAK4 interactors Cdc42 and LIMK1 or the here identified novel PAK4 interactor N-WASP. This may be due to different expression levels in different cell lines, transient and weak interactions, and limitations in peptide ionization and detection by mass spectrometry. Moreover, different activity states of PAK4 may result in partially different PAK4 interactors that could not be detected here. PAK4 may be activated by different stimuli, such as by growth factors, adhesion to the ECM or activation of upstream PAK4 effectors, such as Cdc42 [11, 23, 24, 66]. Such stimuli may also affect the state of the cell and may also affect the PAK4 intracellular localization, factors that could also influence the expected PAK4 interactome.

In summary, the PAK4 interactome provides a valuable resource for future investigations on the role of PAK4 in physiology and disease. We also propose a new mechanism for PAK4 regulation of actin cytoskeleton dynamics.

## MATERIALS AND METHODS

### Cell culture and cell lysate preparation

MCF7 stably overexpressing FLAG-PAK4 and FLAG-BAP cell lines were described [24]. These cells were grown with Dulbecco's Modified Eagle Medium (DMEM) (41965, Life Technologies) supplemented with 10% fetal bovine serum (FBS)(10270, Life Technologies) and 150 μg/ml G418 (11811, Life Technologies) at 37°C with 5% CO_2_. For the WC, cell were lysed with buffer containing 50 mM HEPES, 150 mM NaCl, 5 mM MgCl_2_, 1 mM EDTA, 10% Glycerol, 1% Chaps and freshly added protease inhibitors (1697498, Roche) and phosphatase inhibitors (P0044, Sigma). For Cyt and Nuc fraction, cells were lysed with cytoplasmic buffer containing 10 mM HEPES, pH 7.5, 10 mM KCl, 1.5 mM MgCl_2_, freshly added protease/phosphatase inhibitors, incubated on ice for 15 min, homogenized by 30 strokes with kontes dounce pestle B and checked by microscope, 95% of the cell displayed trypan blue (T8154, Sigma) staining. Nuclei were pelleted by centrifugation at 350 g for 5 min at 4°C, and the supernatant was the cytoplasmic lysate. The pellet was washed with cytoplasmic buffer 3 times, then dissolved in nuclear buffer containing 20 mM HEPES, 25% glycerol, 0.42 M NaCl, 1.5 mM MgCl_2_, 0.2 mM EDTA, freshly added protease/phosphatase inhibitors and kept on ice for 30 min before vortexing several times, then cleared at 16.000 g for 20 min at 4°C to receive the nuclear fraction. All lysates were stored at -80°C. Protein concentrations were measured in triplicates using a BCA protein assay kit (#23228, Thermo Scientific).

### Immunoprecipitation

1500 μg protein lysate from MCF7 stably overexpressing FLAG-PAK4 and FLAG-BAP was pre-cleared with 150 μl protein G plus agarose (sc-2002, Santa Cruz), and 3 μg mouse IgG (I5381, Sigma) for 2 h at 4°C, the pre-cleared lysates were centrifuged and carefully transferred to a new tube and 30 μl anti-FLAG M2 affinity gel (F2426, Sigma) was added, and then gently rotated overnight at 4°C. Samples were washed three times with a buffer containing 10 mM HEPES, pH 7.5, 10 mM KCl, 1.5 mM MgCl_2_ with 0.5% Chaps and three times without Chaps, eluted with 100μl buffer containing 50 mM HEPES, 150 mM NaCl, 15 μg 3×FLAG peptides (F4799, Sigma) for 1h at 4°C. Samples were stored at -80°C.

### Immunoblotting

Samples were separated on SDS-polyacrylamide gels and transferred to an Immobilon-P Membrane (IPVH00010, Millipore). After blocking for 1 h at room temperature with 5% no-fat milk (A0830, Applichem), the membranes were incubated with primary antibodies overnight at 4 °C. Antibodies for ARPC2 (HPA008352) were obtained from Atlas Antibodies. 14-3-3ε (CPCT-YWHAE-1), 14-3-3 α/β (CPCT-YWHAB-1) antibodies were obtained from DSHB, pRb antibody (554136) from BD-Pharmingen and vinculin antibody (ab11194) from Abcam, and the PAK4 pab 6508 were generated in our laboratory [31]. The membranes were then incubated with a horseradish peroxidase-conjugate secondary antibody (mouse: 715-035-150, Rabbit: 111-035-144, Jackson ImmunoResearch) for 1 h at room temperature. Membranes were developed by enhanced chemiluminescence (#32106, Thermo Science).

### Sample preparation for mass spectrometry

Four biological replicates from whole cell, cytoplasmic and nuclear fractions were immunoprecipitated and eluted as described above. Samples were mixed with 1 mM DTT, 8 M urea, 25 mM HEPES, pH 7.6 in a centrifugation filtering unit (Nanosep® Centrifugal Devices with Omega™ Membrane, 10 kDa cutoff), and centrifuged for 15 min at 14.000 g, followed by another addition of the 8 M urea buffer and centrifugation. Proteins were alkylated by 55 mM IAA, in 8 M urea, 25 mM HEPES, pH 7.6 for 10 min, centrifuged, followed by 2 more additions and centrifugations with 8 M urea, 25 mM HEPES pH 7.6. Trypsin (Promega). 1:50, trypsin:protein, was added to the samples in 0.250 M urea, 25 mM HEPES and digested overnight at 37 °C. The filter units were centrifuged for 15 min at 14.000 g, followed by another centrifugation with MQ. Flow-throughs of peptides were collected and iTRAQ (isobaric Tags for Relative and Absolute Quantitation) labeled (PAK4, 114, 116, 118, 121 and BAP 113, 115, 117, 119) according to the manufacturer's instructions, pooled, and cleaned by a strata-X-C-cartridge (Phenomenex). Peptides were dried by speedvac and dissolved in 3% acetonitrile (ACN), 0.1 % formic acid before analysis on MS.

### LC-ESI-LTQ-Orbitrap analysis

Before analysis on the LTQ Orbitrap Velos (Thermo Fischer Scientific,), peptides were separated using an Agilent 1200 nano-LC system. Samples were trapped on a Zorbax 300SB-C18, and separated on a NTCC-360/100-5-153 (Nikkyo Technos Ltd) column using a gradient of A (3% ACN, 0.1% FA) and B (95% ACN, 0.1% FA), ranging from 3 % to 40% B in 120 or 240 min with a flow of 0.4 μl/min. The LTQ Orbitrap Velos was operated in a data dependent manner, selecting 5 precursors for sequential fragmentation by CID and HCD (Higher-energy Collisional Dissociation), and analyzed by the linear iontrap and orbitrap, respectively. The survey scan was performed in the Orbitrap at 30.000 resolution (profile mode) from 300-2000 *m/z,* using lock mass at *m/z* 445.120025, with a max injection time of 500 ms and AGC set to 1 × 10^6^ ions. For generation of HCD fragmentation spectra, a max ion injection time of 500 ms and AGC of 5 × 10^4^ were used before fragmentation with 37,5% normalized collision energy. For FTMS (Fourier Transform Mass Spectrometry) MS2 spectra, normal mass range was used, centroiding the data at 7500 resolution. Peptides for CID were accumulated for a max ion injection time of 200 ms and AGC of 3 × 10^4^, fragmented with 35% collision energy, wideband activation on, activation q 0.25, activation time 10 ms before analysis at normal scan rate and mass range in the linear iontrap. Precursors were isolated with a width of 2 *m/z* and put on the exclusion list for 90 s. Single and unassigned charge states were rejected from precursor selection. The mass spectrometry proteomics data have been deposited to the ProteomeXchange with the dataset identifier PXD004086.

### Peptide and protein identification

All Orbitrap data was searched by SequestHT under the software platform Proteome Discoverer 1.4 (Thermo) against the Uniprot human database (2014-04-07) and filtered to a 1% FDR. A precursor mass tolerance of 15 ppm, and product mass tolerances of 0.02 Da for HCD-FTMS and 0.36 Da for CID-ITMS (Ion trap mass spectrometry) were used. Further settings used were: trypsin with 2 missed cleavage; iodoacetamide on cysteine, iTRAQ on lysine and N-terminus as fixed modifications, and oxidation of methionine as variable modification. Quantization of iTRAQ8 plex reporter ions was done by Proteome Discoverer on HCD-FTMS tandem mass spectra using an integration window tolerance of 20 ppm. Only unique peptides in the data set were used for quantification [67]. To identify PAK4 interactors, we used the fact that interactors will be enriched following IP-MS from PAK4 overexpressing cells in comparison to BAP expressing cell. Because the antibody is the same for both PAK4 and BAP we can assume that all binding to BAP is non-specific and enrichment in PAK4 pulldowns indicate interactors. To define PAK4 pull down proteins in comparison to BAP we used t-test to compare PAK4 and BAP levels using the four replicates of PAK4 and BAP using the Graphpad Prism 6 software. The t-test was corrected for multiple testing and limited to 5% FDR (p≤0.05). In addition, to complement the t-test cut-off, a fold change cut-off was applied according to the 99.9% confidence interval based on the -BAP signal distribution. This provided the fold-change compared to the average FLAG-BAP signal for each fraction used as cut-off (WC: 1.83; Cyt: 1.50; and Nuc: 1.92). Proteins in the FLAG-PAK4 pool displaying this signal or above were considered PAK4 enriched and therefore potential PAK4 interactors. To define PAK1 interactome, we obtained the PAK1 interactome dataset from the Cell migration gateway database (http://data.cellmigration.org/ms_results/comb_hits2n.cgi?h_expts=n050307n01,b050801b03,b050801b02). From this dataset, we excluded those proteins that displayed an interaction with the used FLAG negative control and then applied a cut-off using only proteins with an E-score ≥6, as suggested in this dataset.

### Bioinformatic analysis

To determine the biological processes enrichment in the PAK4 interactome, we employed Cytoscape 3.3 [68] and its plugin BiNGO 2.44 [69], and used whole annotation as reference set and a hypergeometric test with Benjamini-Hochberg false discovery rate correction. Cellular component was analyzed. Furthermore, enrichment analysis of Pfam domain families [70] were performed using program DAVID 6.7 [71]. The default human proteome was used as background list. The significance of the enrichment was based on the Modified Fisher's exact test (EASE score).

The PAK4 interactome were searched against the STRING database, version 10 [72], for protein-protein interactions, where 305 of the 313 PAK4 interacting proteins were found. Only experimental and database confirmed protein-protein interactions were selected and a confidence score ≥0.4 was used. The networks of protein complexes were generated this way and then visualized by Cytoscape 3.3. The PAK4 interaction network was further analyzed for protein clusters using AutoAnnotate, AutoAnnotate is part of the plug-in toolkit in Cytoscape.

### Protein-protein interaction assay

H1299 cell were transfected with an EGFP vector or an EGFP-PAK4 vector by Lipofectamine 3000. Cells were harvested 48h after transfection and lysed with NP-40 cell lysis buffer containing 50 mM Tris-HCl pH7.5, 150 mM NaCl, 5 mM MgCl_2_, 1 mM EDTA, 10% Glycerol, 1% NP-40, and freshly added protease inhibitors (1697498, Roche) and phosphatase inhibitors (P0044, Sigma). 1000 μg protein lysate were immunoprecipated by GFP-Trap (gta-20, ChromoTek), CCTε and N-WASP antibodies with rabbit IgG as control, separated and probed with rat CCTε antibody (MCA2178, BIO-RAD), rabbit ARPC2 (HPA008352, Atlas Antibodies) and rabbit N-WASP antibody (HPA005750, Atlas Antibodies) and mouse GFP antibody (MAB2510, Milipore). For endogenous protein-protein interactions, 1000 μg protein lysate from MCF7 or H1299 cells were immunoprecipated by a rabbit anti-PAK4 antibody (6508) or by a rabbit anti-N-WASP antibody (HPA005750, Atlas Antibodies) using rabbit IgG as control. The immunoprecipitations were, separated on SDS-PAGE, blotted and probed with rabbit anti-N-WASP or anti-PAK4 antibodies.

### *In vitro* kinase assay

HIS-PAK4, GST-PAK4 kinase domain (GST-PAK4 KD (286-591)) and GST-RAF1 (332-344) fusion proteins were produced as described [61]. *In vitro* protein phosphorylation assays were performed as described [24]. Briefly, phosphorylation reactions were incubated a kinase reaction buffer (50 mM HEPES, pH 7.5, 10 mM MgCl_2_, 2 mM MnCl_2_, 0.2 mM dithiothreitol) in the presence of 30 μM ATP and 10 μCi of [γ-32P] ATP and in the presence of the kinase (7 μg of HIS-PAK4 or GST-PAK4 KD) and 2 μg of the substrate (Arp2/3 complex, GST-RAF1 (332-344), or GST-VCA) for 30 min at 30°C. GST-VCA (#VCG03) and Arp2/3 complex (# RP01P) were obtained from Cytoskeleton, Inc. The reaction was stopped by adding sample loading buffer and heating at 95°C for 5 min. Samples were separated by 10% SDS-PAGE and visualized by autoradiography with the PhosphorImager system (Molecular Imager FX, Bio-Rad). GST-RAF1 (332-344) and GST-VCA kinase activity also be detected with phospho-site specific antibodies WASP pSer483/Ser484 (NB100-2307, Novusbio) and RAF1 pSer338 (#9427, Cell Signaling).

### Pull-down assay

3 μg HIS-PAK4 and 3 μg Arp2/3 complex or 3 μg HIS-PAK4 and 3 μg GST-VCA were incubate with 20 μl Ni-NTA Agarose 30 min in 60 μl buffer (50 mM HEPES, pH 7.5, 150 mM NaCl, 5 mM MgCl_2_, 20 mM imidazole and 1 mM ATP) at RT. The supernatant was separated by centrifugation for 10 min at 1200 rpm and the pellet washed 3× with the same buffer. Samples were separated by 10% SDS-PAGE and visualized by silver staining.

### Actin polymerization assay

Pyrene muscle actin (#AP05), GST-VCA and Arp2/3 complex were obtained from Cytoskeleton, Inc. Actin polymerization assay was performed essentially as described [48]. Briefly, all relevant proteins at the indicated concentrations in KMEI (10 mM imidazole, pH 7.0, 50 mM KCl, 1 mM EGTA, and 1 mM MgCl_2_) were incubated 10 min. at RT to allow PAK4 kinase domain phosphorylation. 1.6 μM pyrene labeled Mg-ATP actin in G buffer-Mg (2 mM Tris-HCl, pH 8.0, 0.5 mM DTT, 0.2 mM ATP, 0.1 mM MgCl_2_) was mixed with the indicated samples in KMEI (10 mM imidazole, pH 7.0, 50 mM KCl, 1 mM EGTA, and 1 mM MgCl_2_). Pyrene fluorescence changes (excitation at 360 nm and emission at 405 nm) were followed using a SpectraMAX Gemini EM (Molecular Devices). Polymerization was performed at room temperature.

### siRNA transfection and cytochalasin D treatment

H1299 cells were grown in RPMI-1640 medium (42401, Life Technologies) supplemented with 2 mM glutamine (25030, Life Technologies) and 10% FBS at 37 °C with 5% CO_2_. H1299 cells were transfected with 10 nM siRNA using Lipofectamine RNAiMAX Reagent (13778, Life Technologies) according to the manufacturer's protocol. The control siRNA (1027280) and #1 PAK4 siRNA (SI02660315) were purchased from Qiagen and #2 PAK4 siRNA (5′-CAAGCTGGTGGCCGTCAAGAA -3′) was purchased from GenePharma, Shanghai, China. Cells were harvested at day 3 after transfection. 500 nM Cytochalasin D (C8273, Sigma) was added to the culture media 1 h before harvest.

### G-actin and F-actin Separation

H1299 cells were harvested at day 3 after siRNA transfection, lysed with actin stabilization buffer (50 mM PIPES at pH 6.9, 50 mM NaCl, 5 mM MgCl_2_, 5 mM EGTA, 5% glycerol, 0.1% NP40, 0.1% Triton X-100, 0.1% Tween 20, 0.1% β-mercaptoethanol, 1 mM ATP, protease inhibitor) at 37°C for 10 min, centrifuged at 350 g to pellet unbroken cells and tissue debris, supernatants were further centrifuged at 100,000 g at 4°C for 1h. The supernatant enriched in G-actin was recovered, and the pellet enriched in F-actin was solubilized with actin depolymerization buffer (0.1 M PIPES, pH 6.9, 1 mM MgSO_4_, 10 mM CaCl_2_, and 5 μM cytochalasin D) on ice for 1 h. Aliquots of supernatant and pellet fractions were separated on 10% SDS-PAGE gels and immunoblotted with anti-β-actin (JLA20) antibody from DSHB.

### Immunofluorescence microscopy

MCF7 cell line stably overexpressing FLAG-PAK4 were re-plated for 30 min onto 20 μg/ml collagen type I coated dishes in adhesion buffer (RPMI 1640, 2 mM CaCl_2_, 1 mM MgCl_2_, 0.2 mM MnCl_2_ and 0.5% BSA). Cells were fixed with 3 % paraformaldehyde for 10 min, permeabilized with 0.1% Triton-X for 5 min, blocked with 2 % BSA for 1 h at room temperature and incubated with primary antibodies overnight at 4 °C. Anti-FLAG mab (F3165) were obtained from Sigma. The anti-N-WASP (HPA005750) antibody was obtained from Atlas Antibodies. F-actin was labeled with siR-actin at 1 μM for 1 h (SC001, Cytoskeleton, Inc) and nuclei were stained with 8 μM Hoechst (14533, Sigma). For cell morphology, H1299 cells were attached overnight on a vitronectin (10 μg/ml) coated dish 3 d after siRNA transfection. Cells were fixed, permeabilized and blocked at room temperature. The nuclei were stained with 8 μM Hoechst (14533, Sigma) and F-actin with 0.2 μM Alexa Fluor 568 conjugated phalloidin for 30 min (A12380, Life Technologies). Images were acquired with a Nikon A1 confocal microscope using an oil immersion objective (60X/1.4 NA) and the NIS software.

## SUPPLEMENTARY MATERIALS FIGURES AND TABLES









## Abbreviations

Arp2/3 complex: actin-related protein 2/3 complex
BAP: bacterial alkaline phosphatase
CCT complex: chaperonin containing TCP-1 complex
Cyt: cytoplasmic fraction
F-actin: filamentous actin
FDR: false discovery rate
G-actin: globular actin
GO: gene ontology
IP: immunoprecipitation
iTRAQ: isobaric tags for relative and absolute quantitation
MCM complex: minichromosome maintenance complex
MS: mass spectrometry
Nuc: nuclear fraction
PAK4: p21-activated kinase 4
QMS: quantitative mass spectrometry
WC: whole cell
