# Supplementary material for: Identification of the PAK4 interactome reveals PAK4 phosphorylation of N-WASP and promotion of Arp2/3-dependent actin polymerization
Source: Oncotarget. 2017 Aug 18;8(44):77061–74. doi: 10.18632/oncotarget.20352 (PMC5652764; doi:10.18632/oncotarget.20352)
Supplement: Supplementary file 1 [file oncotarget-08-77061-s001.pdf]

# Identification of the PAK4 interactome reveals PAK4 phosphorylation of N-WASP and promotion of Arp2/3-dependent actin polymerization

## SUPPLEMENTARY MATERIALS

**Supplementary Table 1: Quantitative mass spectrometry data for the PAK4 interactome.**

**See Supplementary File 1**

**Supplementary Table 2: Complete list of GO cellular components terms enriched in the PAK4 interactome. Red text: selected GO terms in Supplementary Figure 2A.**

**See Supplementary File 2**

**Supplementary Table 3: Complete list of Pfam protein domains enriched in the PAK4 interactome. Red text: selected GO terms in the Supplementary Figure 2B.**

**See Supplementary File 3**

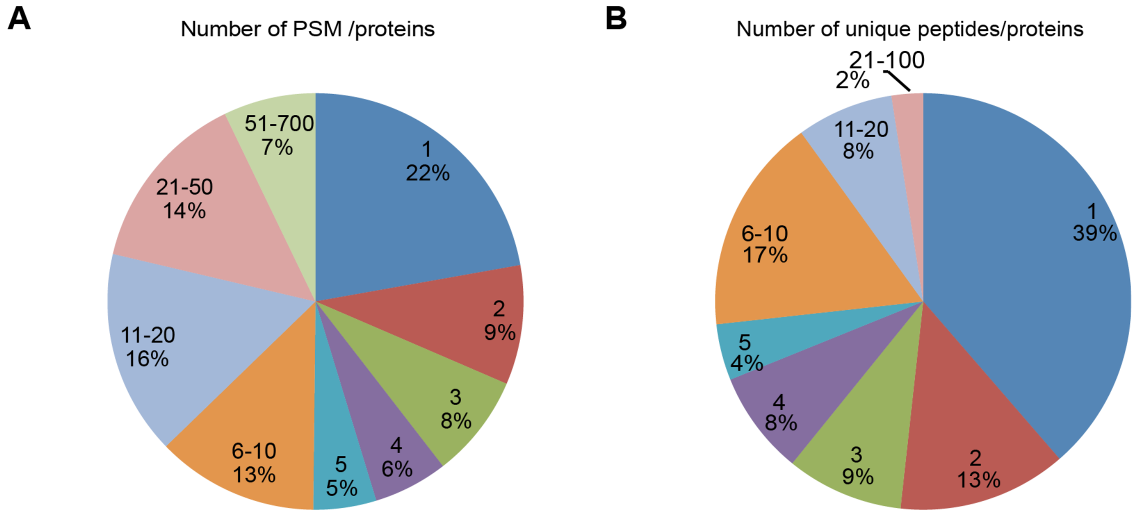


**Supplementary Figure 1: Controls for the quantitative mass spectrometry. (A)** The pie chart shows the percentage of proteins for different peptides spectrum matches per protein. **(B)** The pie chart shows the percentage of proteins with the same number of unique, identified peptides per protein.

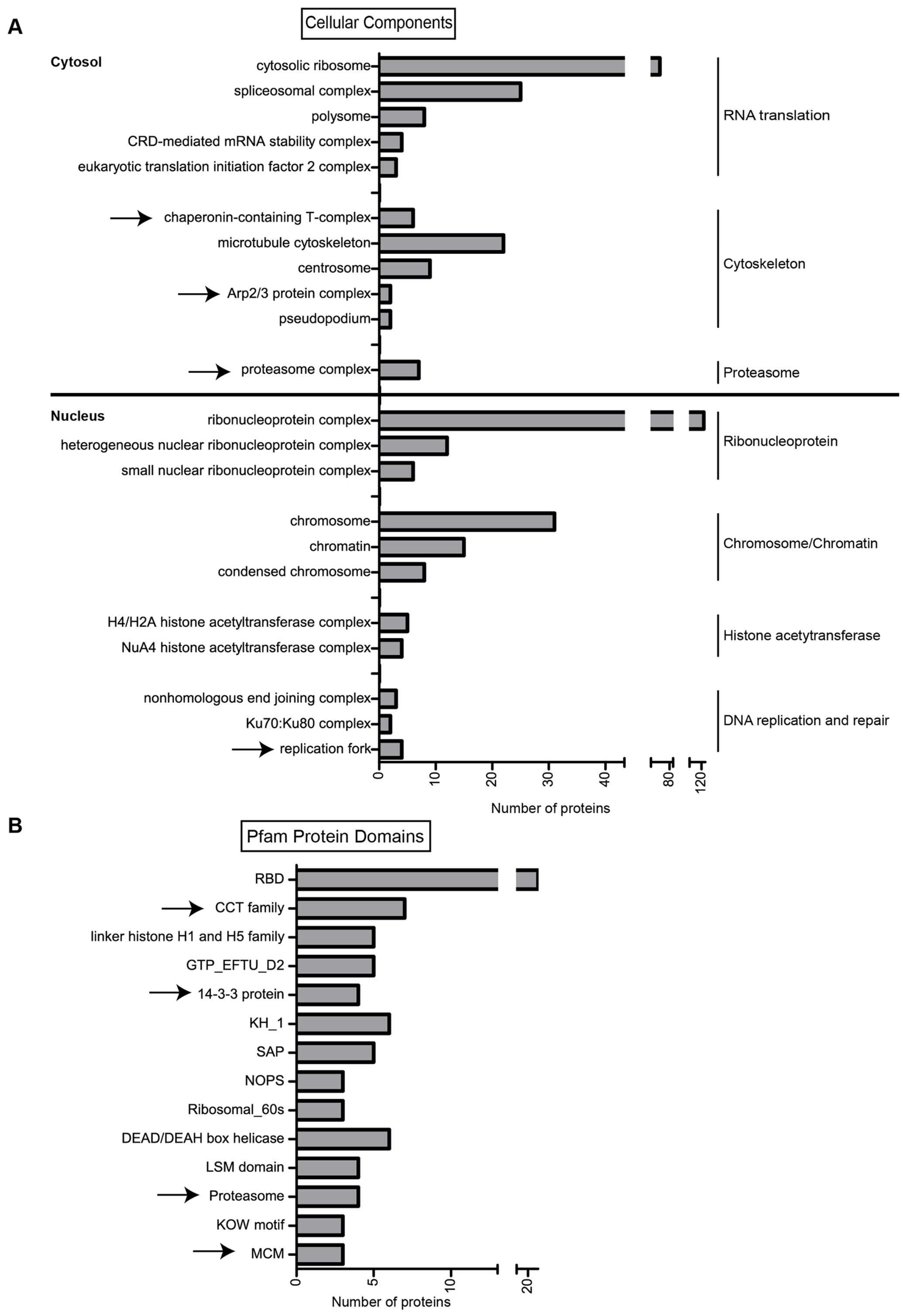


**Supplementary Figure 2: GO term enrichment in the PAK4 interactome. (A)** Cellular components were curated to display biologically relevant GO terms and grouped according to functional similarity. The presented order of main groups (indicated to the right in panel A) are based on P values of enrichment with the lowest P value on top, and the order within the groups is also according to P values enrichment within each group, with the lowest P value on top. **(B)** Pfam protein domains enriched in PAK4 interactome. The presented orders are based on p values of enrichment with the lowest P value on top for each group. All presented GO terms and Pfam domains display $p< 0.05$ for enrichment. All repetitive terms and terms that appeared not to provide useful biological information were removed. Arrows indicate the cellular components or protein domains in agreement with protein clusters identified by the STRING database (Figure 2B).

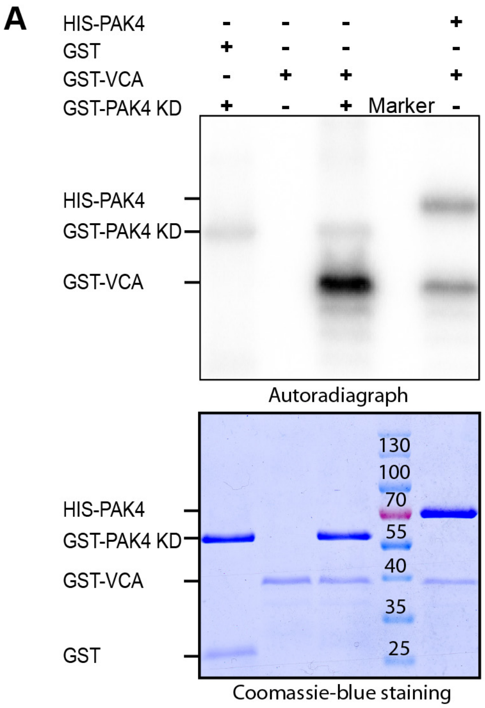


**Supplementary Figure 3: Recombinant PAK4 phosphorylation of the WASP VCA domain. (A)** PAK4 and PAK4 kinase domain (PAK4 KD) mediated phosphorylation of the WASP VCA domain was analyzed by an *in vitro* kinase assay using recombinant HIS-PAK4 and GST-PAK4 KD together with GST-VCA as a substrate, with GST as a negative control (upper panel). The lower panel displays the protein loading in the assay by Coomassie Brilliant Blue staining.
